# Regulation of lipid droplet (LD) formation in hepatocytes *via* regulation of SREBP1c by non-coding RNAs

**DOI:** 10.3389/fmed.2022.903856

**Published:** 2022-09-20

**Authors:** Shereen A. El Sobky, Nourhan K. Aboud, Nihal M. El Assaly, Injie O. Fawzy, Nada El-Ekiaby, Ahmed I. Abdelaziz

**Affiliations:** ^1^School of Medicine, Newgiza University (NGU), Giza, Egypt; ^2^Clinical Chemistry Department, Theodor Bilharz Research Institute, Giza, Egypt

**Keywords:** SREBP1c, mTOR, ncRNAs, NAFLD, *de novo* lipogenesis (DNL)

## Abstract

**Introduction:**

Increased *de novo* lipogenesis (DNL) is one of the key factors contributing to fat accumulation and non-alcoholic fatty liver disease (NAFLD). Among the critical transcription factors (TFs) regulating DNL is mTOR and its downstream lipogenic TF, SREBP1c. In recent years, it has been established that non-coding RNAs (ncRNAs) play role in both biological processes and disease pathogenesis. Our group has previously characterized microRNAs that can target and regulate the expression of both mTOR and SREBP1c. Accordingly, this study aimed to broaden our understanding of the role of ncRNAs in regulating the mTOR/SREBP1c axis to elucidate the role of the non-coding transcriptome in DNL and lipid droplet (LD) formation. Hence, short ncRNA, miR-615-5p, and long non-coding RNA (lncRNA), *H19*, were chosen as they were previously proven to target mTOR by our group and in the published literature, respectively.

**Methodology:**

Huh-7 cells were treated with 800 μM oleic acid (OA) to promote LD formation. Transfection of miR-615-5p mimics or *H19* over-expression vectors was performed, followed by the measurement of their downstream targets, mTOR and SREBP, on the mRNA level by quantitative real-time PCR (qRT-PCR), and on the protein level by Western blot. To determine the functional impact of miR-615-5p and *H19* on LD formation and triglyceride (TG) accumulation, post-transfection LDs were stained, imaged, and characterized, and TGs were extracted and quantified.

**Results:**

miR-615-5p was able to reduce mTOR and SREBP1c significantly on both the mRNA and protein levels compared to control cells, while H19 caused a reduction of both targets on the protein level only. Both miR-615-5p and *H19* were able to significantly reduce the LD count and total area, as well as TG levels compared to control cells.

**Conclusion:**

To conclude, this study shows, for the first time, the impact of miR-615-5p and *H19* on the mTOR/SREBP1c axis, and thus, their functional impact on LDs and TG accumulation. These findings might pave the way for using ncRNAs as potential therapeutic targets in the management of fatty liver.

## Introduction

Increased *de novo* lipogenesis (DNL) is one of the contributing factors leading to fat accumulation and non-alcoholic fatty liver disease (NAFLD) (1). DNL is controlled by lipogenic transcription factors such as sterol regulatory element-binding protein (SREBP), carbohydrate response element-binding protein (ChREBP), and peroxisome proliferator-activated receptor gamma (PPARγ) (2). These transcription factors may contribute to NAFLD as patients with NAFLD have increased DNL that is not suppressed by fasting or high nocturnal levels of free fatty acids (FFAs) in plasma (3). SREBP is the main transcription factor responsible for hepatic DNL by insulin (1). There are three isoforms of SREBP: SREBP1c, SREBP1a, and SREBP2. Despite functional overlap between different SREBPs, SREBP1c is the one which is primarily responsible for fatty acid synthesis and triglyceride (TG) accumulation (2).

Non-coding RNAs (ncRNAs) can be classified according to their length, localization, and function into several classes. They can be classified into small ncRNAs being <200 nucleotides (nts) long and long ncRNAs of more than 200 nts length (4). Among the small ncRNAs are the microRNAs (miRNAs). miRNAs are small single-stranded RNAs of ≈22 nucleotides that were shown to be among the main regulators for gene expression (5). Several miRNAs were reported to affect NAFLD and DNL, such as miR-122 and miR-33, which decrease SREBP expression as well as downstream enzymes needed for lipogenesis (6, 7). On the other hand, our group also found that miR-29a induces SREBP1c expression as well as lipid droplet (LD) and TG accumulation in HCV-infected cell models (8).

lncRNAs, on the other hand, have shown great versatility, regulating almost every step of eukaryotic gene expression through mechanisms such as chromatin remodeling, transcription machinery recruitment, mRNA processing and delivery to the cytoplasm, mRNA stabilization, translation, and posttranslational processing (9). In addition, lncRNAs are also able to fold into secondary and tertiary structures, enabling them to interact with proteins as well (9). Among the lncRNAs affecting DNL is MALAT1, which was found to induce SREBP1c expression as well as hepatic lipid accumulation (10). On the other hand, lncHR1 was found to repress SREBP1c and hepatic lipid accumulation (11). Furthermore, inhibition of PLIN2, a lipid droplet protein member, was reported to markedly increase lncRNA *H19*, resulting in a decrease in liver TGs (12).

Among the regulators of SREBP1c is mTOR (mammalian or mechanistic target of rapamycin), which is another activator of lipogenesis (13–15). mTOR nucleates both mTORC1 and mTORC2 complexes, which were found to activate SREBP1c expression and maturation (14, 16, 17). We have previously shown a tumor-suppressive effect for miR-615-5p in hepatocellular carcinoma by downregulating mTOR (18). Moreover, Wu et al. have also showed that miR-615-5p is capable of targeting mTOR in colorectal cancer (19). Interestingly, mTOR was also found to be regulated by lncRNA *H19* in pituitary tumors (20). In addition, lncRNA *H19* was shown to target the AKT/mTOR pathway in psoriasis (21), as well as hepatic stellate cells and fibrotic mouse models (22). In these previously published studies, miR-615-5p and lncRNA *H19* were validated to target mTOR; however, pathways other than the mTOR/SREBP axis were investigated, and hence, their impact on LD and TG accumulation was not explored. Therefore, this study sought to regulate the lipogenic SREBP1c through its upstream TF, mTOR, *via* these validated ncRNAs, particularly since their impact on lipogenic activity has not been thoroughly investigated previously.

Therefore, the aim of the study is to examine the role of both ncRNAs on lipogenesis by investigating their effect on the lipogenic mTOR/SREBP axis while studying the net impact of this regulation on LD formation and TG accumulation in hepatic cell lines.

## Methodology

### Cell culture

The human hepatocellular carcinoma cell line (Huh-7) was provided by Professor Kai Breuhahn (University of Heidelberg, Germany) and was cultured in Dulbecco's modified Eagle medium (DMEM) supplemented with 4.5 g/L glucose, L-glutamine (Biowest, France), 10% fetal bovine serum (Lonza, Switzerland), and 1% streptomycin/penicillin (Biowest, France). Cells were incubated under normal conditions (37°C and 5% CO_2_).

### MTT assay

A total of 10,000 Huh-7 cells were seeded in 96-well plates and treated with different concentrations of oleic acid (OA) (Sigma Aldrich, United States) (250 μM, 500 μM, 1000 μM). 1X PBS was used as a negative control. MTT assay was performed 24 h and 48 h posttreatment. Absorbance was measured at 570 nm.

### Transfection of oligonucleotides or plasmids

The Huh-7 cells were seeded in 24-well plates (75,000 cells/well) and then treated with 800 μM OA the following day (this concentration reflects the fasting plasma-free FA levels of patients with NASH) (23). After 24 h, the cells were transfected with either miR-615-5p mimics or negative control oligonucleotides (NC) (Qiagen, Germany) using HiPerFect transfection reagent (Qiagen, Germany) following the manufacturer's instruction. For plasmid transfection, the same procedure was followed but using SuperFect transfection reagent (Qiagen, Germany) to transfect *H19* over-expression plasmid (provided by Prof. Kiemer, Saarland University, Germany) or empty vector.

### Reverse transcription and qRT-PCR

Total cellular RNA was extracted 48 h post-transfection using TRIzol reagent (Invitrogen, USA) following the manufacturer's instructions. Total RNA was then DNAse-digested (NEB, USA) and reverse-transcribed into single-stranded complementary DNA (cDNA) using the high-capacity cDNA archive kit (Applied Biosystems, USA) following the manufacturer's instruction. The mRNA expression levels were quantified using TaqMan gene assays for *H19*, mTOR, and SREBP1c (Applied Biosystems, USA). On the other hand, miRNA was reverse-transcribed and quantified using the TaqMan microRNA Reverse Transcription Kit (Applied Biosystems, USA) and specific primers for hsa-miR-615-5p (Assay ID: 002353) or RNU6B (Assay ID: 001093). The mRNA and miRNA expression levels were quantified using a StepOne Real-Time PCR instrument (Applied Biosystems, USA). Relative expression was calculated using the comparative CT method. Beta-actin was used as an internal control for cellular genes, while RNU6B was used as an internal control for miRNAs.

### Western blot

Proteins were extracted 48 h post-transfection using TRIzol reagent (Invitrogen, USA) following the developed protocol by Ashley et al. (24). Proteins were quantified using a Modified Lowry Protein Assay Kit (Pierce Biotechnology, Inc.) following the manufacturer's instructions. A measure of 100 μg of proteins were separated using 8% SDS–PAGE. Proteins were transferred into a nitrocellulose membrane, and the expression of proteins was detected using mouse anti-mTOR (1:250) (4517, Cell signaling, USA), mouse anti-SREBP1c (2A4) antibody (1:50) (sc-13551, Santa Cruz Biotech, USA), and mouse anti-β-actin (C4) antibody (1:1000) (sc-47778, Santa Cruz Biotech, USA), respectively. Anti-mouse IgG-HRP (sc-516102, Santa Cruz Biotech, USA) was used along with the Clarity™ Western ECL Substrate (Biorad, USA) for detection of the protein bands. Blots were imaged using ImageQuant LAS500 (GE Healthcare Bio-Sciences, Sweden) and analyzed using ImageJ software (http://rsbweb.nih.gov/ij).

### Lipid droplet staining, imaging, and quantification

Lipid droplets were stained 48 h post-transfection as follows: cultured cells were fixed with 4% formaldehyde in PBS for 10 min. The cells were then washed three times with PBS, followed by permeabilization with 0.05% Tween 20 in PBS. The cells were washed three times with 1X PBS and then treated with 60% isopropanol for 5 min. This was followed by the addition of diluted oil red-O for 20 min (Serva, Germany). After removal of oil red-O, the cells were washed three times with distilled water. For imaging of lipid droplets, hematoxylin was added after washing oil red-O with distilled water, left to stand for 1–3 min, and then washed under running tap water to change its color from purple to blue. Lipid droplets were then imaged at 40x (Axiom Zeiss, Germany). For measuring oil red-O absorbance, oil red-O was eluted in 100% isopropanol and measured at 500 nm.

Digital analysis of lipid droplets from the cells stained with oil red-O was carried out, and imaged as suggested by Deutsch et al. method with some modifications. After staining lipid droplets with oil red-O and nuclei with hematoxylin, five random pictures at 40× were captured and analyzed using ImageJ software (http://rsbweb.nih.gov/ij). Brightness/contrast for each picture was adjusted to enhance contrast between oil red-O and bluish hematoxylin, and then images were color-thresholded for the color saturation of lipid droplet signals with the command option “pass”. Masked images were then compared with original images. After setting the scale for images, count, total area, and average size of lipid droplets were quantified. For each picture, these data were divided by the number of cells in the picture. Each experiment was normalized to itself.

### Triglyceride (TG) extraction and quantification

Triglycerides were extracted 48 h post-transfection using the Folch extraction method (25, 26) as follows: cells were collected and homogenized in 1.5 ml chloroform:methanol (2:1) and left on ice for 30 min with occasional vortexing. This was followed by the addition of 0.38 ml water, and the mixture was left for 10 min on ice. The mixture was then centrifuged at 400xg for 5 min. The lower TG layer was collected and evaporated under vacuum using an Eppendorf Concentrator plus (Eppendorf, Germany), and then TGs were dissolved in 5% NP-40 and quantified using a triglyceride quantification colorimetric/fluorometric kit (MAK266, Sigma Aldrich, United States) following the manufacturer's instructions. The protein layer found in the interphase was collected and quantified using modified Lowry protein assay (Applied biosystem, USA) following the manufacturer's instructions. For each sample, the TG concentration was normalized to its respective protein level.

### Statistical analysis

Gene expression was expressed in relative quantitation (RQ). miRNA or gene expression was compared using Student's unpaired *t*-test. The normality test was run for all results obtained from each experiment. The unpaired *t*-test or Mann–Whitney was used for *p*-value calculation depending on data distribution. A *p*-value <0.05 was considered statistically significant. ^***^ = *P* < 0.001, ^**^ = *P* < 0.01, ^*^ = *P* < 0.05, and ns = statistically not significant. All data were the result of multiple independent experiments. All data were statistically analyzed using GraphPad Prism 5.00 software and presented as mean ± standard error of the mean SEM.

## Results

### Determination of viability of Huh-7 cells upon exposure to different OA concentrations

**Figure 1:**
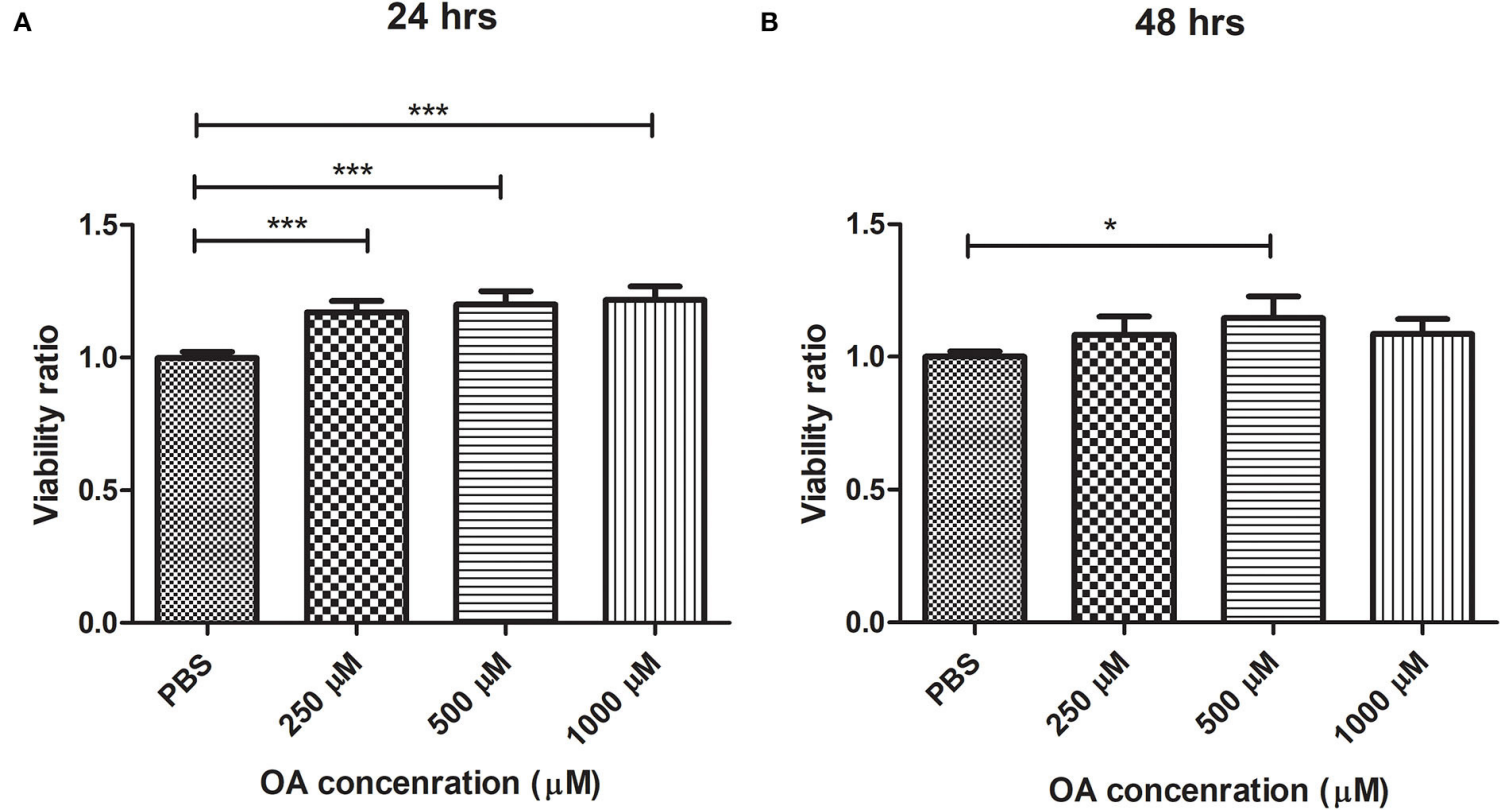
Determining Huh-7 cell viability upon exposure to different concentrations of oleic acid. At 24 h **(A)**, Huh-7 cell viability increased significantly at 250 μM (*p* = 0.0006), 500 μM (*p* = 0.0004), and 1000 μM (*p* = 0.0002) compared to PBS (*n* = 2, pentaplicate), whereas at 48 h, **(B)** Huh-7 cell viability only increased significantly at 500 μM (*p* = 0.0396), but there was no significant change with other concentrations (*n* = 2, pentaplicate). The unpaired two-tailed *t*-test was performed. *** *P* < 0.001, *P < 0.05.

The potential cytotoxic effect of different concentrations of OA on Huh-7 cells was determined using MTT assay at 24 and 48 h. At 24 h, Huh-7 cell viability had significantly increased at the following concentrations: 250 μM (*p* = 0.0006, 1.171 ± 0.04183), 500 μM (*p* = 0.0004, 1.201 ± 0.04998), and 1000 μM (*p* = 0.0002, 1.217 ± 0.05147) compared to cells treated with PBS (*n* = 2, pentaplicate) (Figure 1A), whereas at 48 h, there was no significant change between cell viability at 250 μM or 1000 μM compared to PBS (Figure 1B). However, the cells exhibited a significant increase in viability at 500 μM (*p* = 0.0396, 1.147 ± 0.08112) compared to PBS at 48 h (Figure 1B) (*n* = 2, pentaplicate).

### Validating the lipogenic effect of 800 μM OA on Huh-7 cells

**Figure 2:**
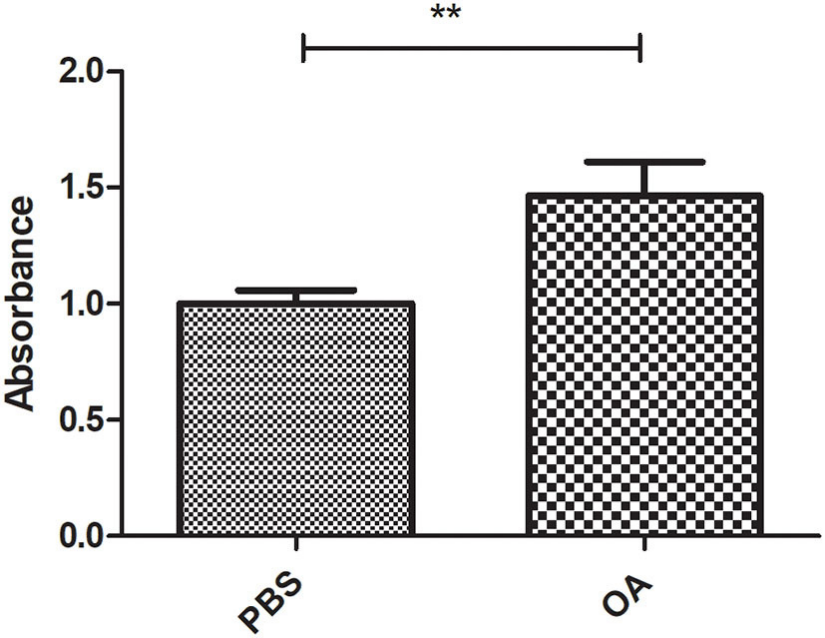
Validating the lipogenic effect of 800 μM on Huh7 cells. Oil red-O absorbance of LDs from cells treated with 800 μM OA showed significant increase (*p* = 0.0074) compared to cells treated with PBS (*n* = 3, quadruplicate). The unpaired two-tailed *t*-test was performed. **P < 0.01.

To confirm the lipogenic effect of 800 μM OA on Huh-7 cells [the concentration of fasting plasma-free FA of patients with NASH (23)], LDs were stained after treatment with PBS or 800 μM OA. LDs of cells treated with 800 μM OA showed a significant increase in the oil red-O absorbance (*p* = 0.0074, 1.465 ± 0.1460) compared to the cells treated with PBS (1.000 ± 0.05947) (*n* = 3, quadruplicate) (Figure 2).

### Expression profile of miR-615-5p and LncRNA *H19* in cells treated with 800 μM OA compared to controls

**Figure 3:**
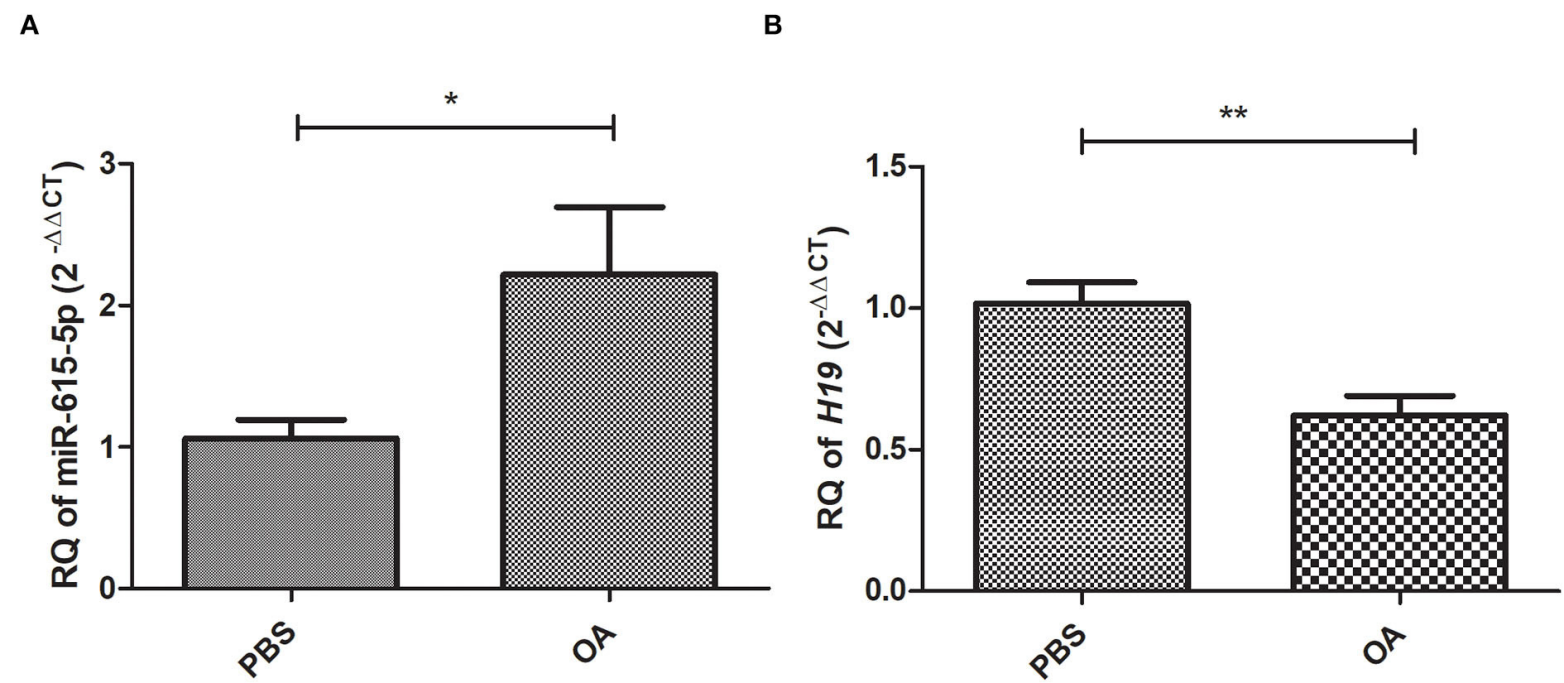
Expression profile of miR-615-5p and *H19* in cells treated with 800 μM compared to controls treated with PBS. Expression pattern of miR-615-5p and *H19* was determined in Huh-7 cells treated with 800 μM compared to control. **(A)** miR-615-5p levels were upregulated in cells treated with OA compared to controls (*p* = 0.0339) (*n* > 3, duplicate). **(B)** lncRNA *H19* levels were downregulated in cells treated with OA compared to controls (*p* = 0.0019) (*n* > 3, duplicate). The unpaired two-tailed *t*-test was performed. **P < 0.01, *P < 0.05.

Relative expression of miR-615-5p and lncRNA *H19* was investigated in cells treated with 800 μM OA compared to control cells treated with PBS. miR-615-5p was found to be upregulated (*p* = 0.0339, 2.215 ± 0.4799) (Figure 3A) in cells treated with 800 μM OA compared to control (1.061 ± 0.1308) (*n* > 3, duplicate). On the other hand, *H19* was found to be downregulated (*p* = 0.0019, 0.6216 ± 0.06853) in cells treated with 800 μM OA compared to control (1.017 ± 0.07524) (Figure 3B) (*n* > 3, duplicate).

### Expression profile of lipogenic transcription factors mTOR and SREBP1c in cells treated with 800 μM OA compared to controls

**Figure 4:**
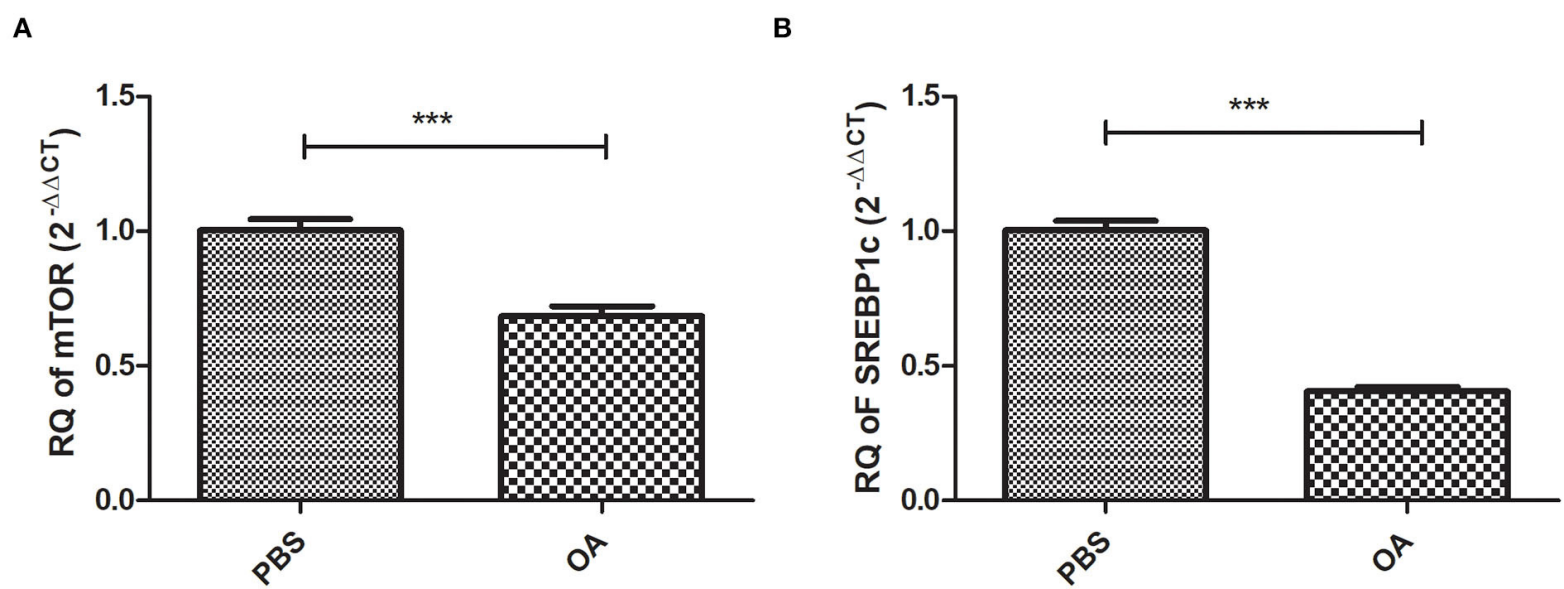
Expression profile of lipogenic transcription factors: mTOR and SREBP1c in cells treated with 800 μM compared to controls treated with PBS. Expression profile of mTOR and SREBP1c was determined in Huh-7 cells treated with 800 μM compared to control. Both mTOR **(A)** (*p* = 0.0002) and SREBP1c **(B)** (*p* = <0.0001) mRNA levels were downregulated in cells treated with OA compared to controls (*n* = 3, duplicate). The unpaired two-tailed *t*-test was performed. ***P < 0.001.

Relative expression of mTOR and SREBP1c at the mRNA level was examined in cells treated with 800 μM OA compared to control cells treated with PBS. Both mTOR (*p* = 0.0002, 0.6842 ± 0.03691) and SREBP1c (*p* = <0.0001, 0.4057 ± 0.01724) were found to be downregulated in cells treated with 800 μM OA compared to control (1.003 ± 0.03963) (1.003 ± 0.03614), respectively (*n* = 3, duplicate) (Figures 4A,B, respectively).

### Impact of manipulating miR-615-5p expression on mRNA levels of lipogenic transcription factors mTOR and SREBP1c

**Figure 5:**
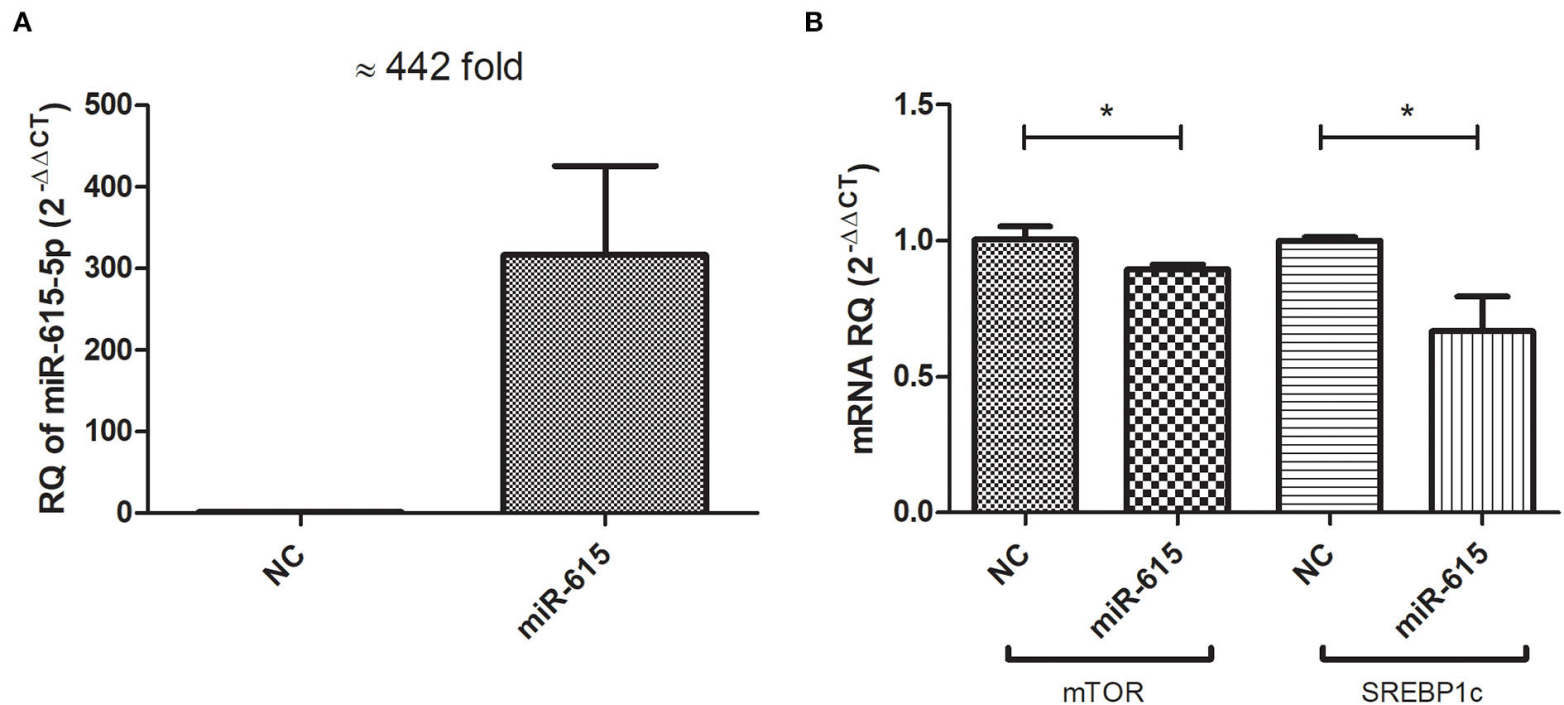
Impact of miR-615-5p on lipogenic transcription factors: mTOR and SREBP1c mRNA levels. Cells transfected with miR-615-5p showed a mean of 442-fold increase (*p* = 0.0195) compared to cells transfected with NC, thus confirming efficient transfection **(A)**. Huh-7 cells treated with 800 μM OA, followed by miR-615 transfection showed a significant decrease in the mRNA level of mTOR (*p* = 0.0429) and SREBP1c (*p* = 0.0457) concomitantly (*n* > 3, duplicate) **(B)**. The unpaired two-tailed *t*-test was performed. *P < 0.05.

Huh-7 cells were treated with 800 μM OA, and on the next day, the cells were transfected with miR-615-5p oligonucleotides or negative control oligonucleotides with no homology to mammalian genes (NC). Efficient transfection was then confirmed by measuring the relative expression of miR-615-5p in mimicked cells relative to cells transfected with NC. Mimicked cells showed a mean 442-fold increase in miR-615-5p expression (*p* = 0.0195) compared to NC (Figure 5A). Mimicking of miR-615-5p in Huh-7 cells treated with 800 μM OA resulted in a decrease in the mRNA level of mTOR (*p* = 0.0429, 0.8945 ± 0.01901) compared to NC (1.006 ± 0.04784) (*n* > 3, duplicate) (Figure 5B). Concomitantly, SREBP1c was also reduced on the mRNA level in cells mimicked with miR-615-5p (*p* = 0.0457, 0.6671 ± 0.1279) compared to NC (1.000 ± 0.01217) (*n* > 3, duplicate) (Figure 5B).

### Impact of forcing the expression of miR-615-5p on mTOR and SREBP protein levels

**Figure 6:**
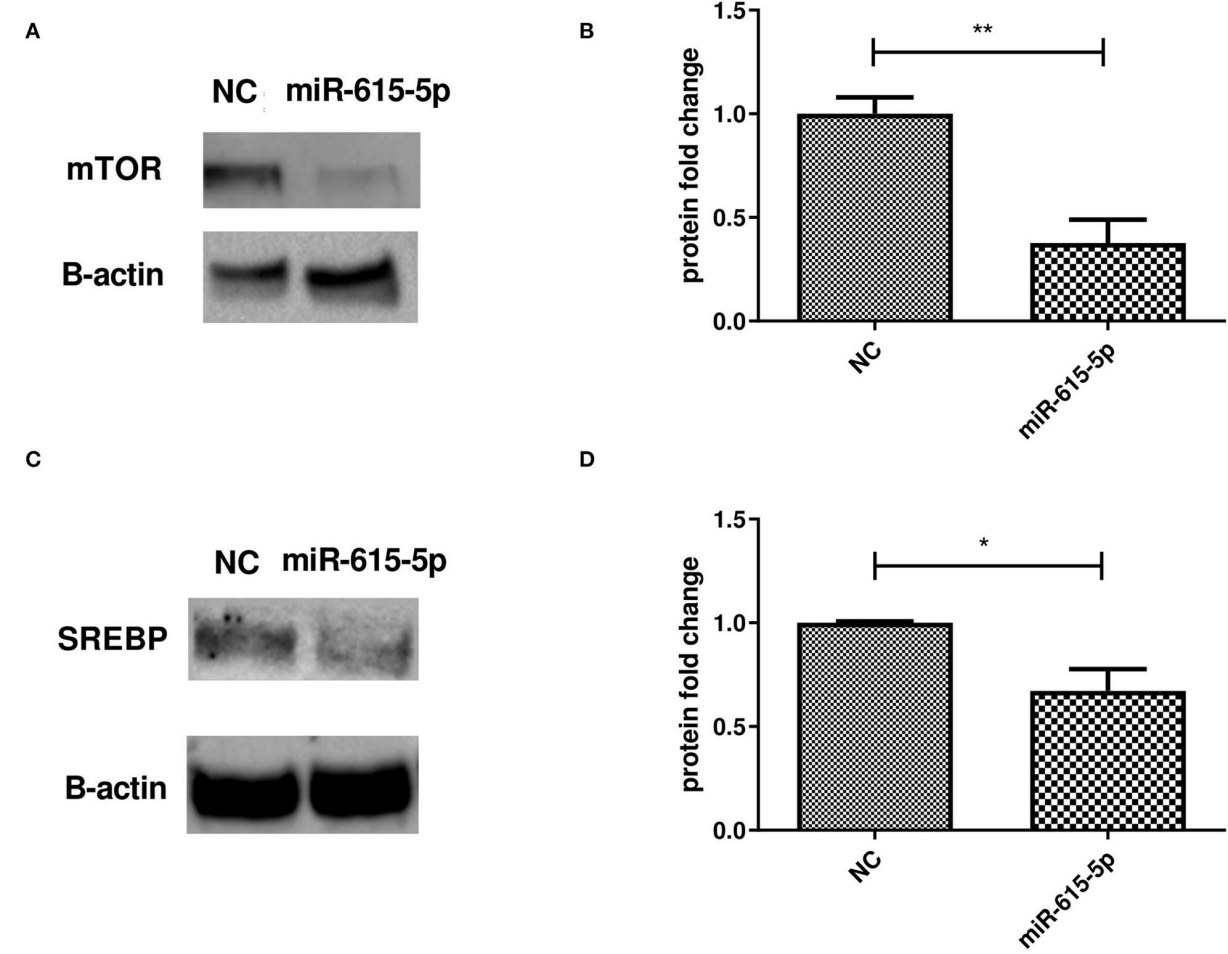
Impact of miR-615-5p on mTOR and SREBP protein levels. Determining the impact of forcing the expression of miR-615-5p in Huh-7 cells treated with 800 μM OA on mTOR/SREBP1 protein levels. **(A)** Representative figure of mTOR blots showing a decrease in the protein level of mTOR. **(B)** Relative quantification of the mTOR protein level in cells treated with an miR-615 mimic and NC (*p* = 0.0020) (*n* = 3). **(C)** Representative figure of SREBP1 blots showing a decrease in the protein level of SREBP1. **(D)** Relative quantification of the SREBP1 protein level in cells treated with an miR-615 mimic and NC (*p* = 0.0146) (*n* > 3). The unpaired two-tailed *t*-test was performed. **P < 0.01, *P < 0.05.

Forcing the expression of miR-615-5p in Huh-7 cells treated with 800 μM OA resulted in a decrease in the protein level of mTOR (*p* = 0.0020, 0.3763 ± 0.1131) compared to NC (1.000 ± 0.07920) (*n* = 3) (Figures 6A,B). This was accompanied by a reduction in SREBP1c protein levels and in cells mimicked with miR-615-5p (*p* = 0.0146, 0.6717 ± 0.1056) compared to NC (1.000 ± 0.006537) (*n* > 3) (Figures 6C,D).

### Impact of miR-615-5p on lipid droplets

**Figure 7:**
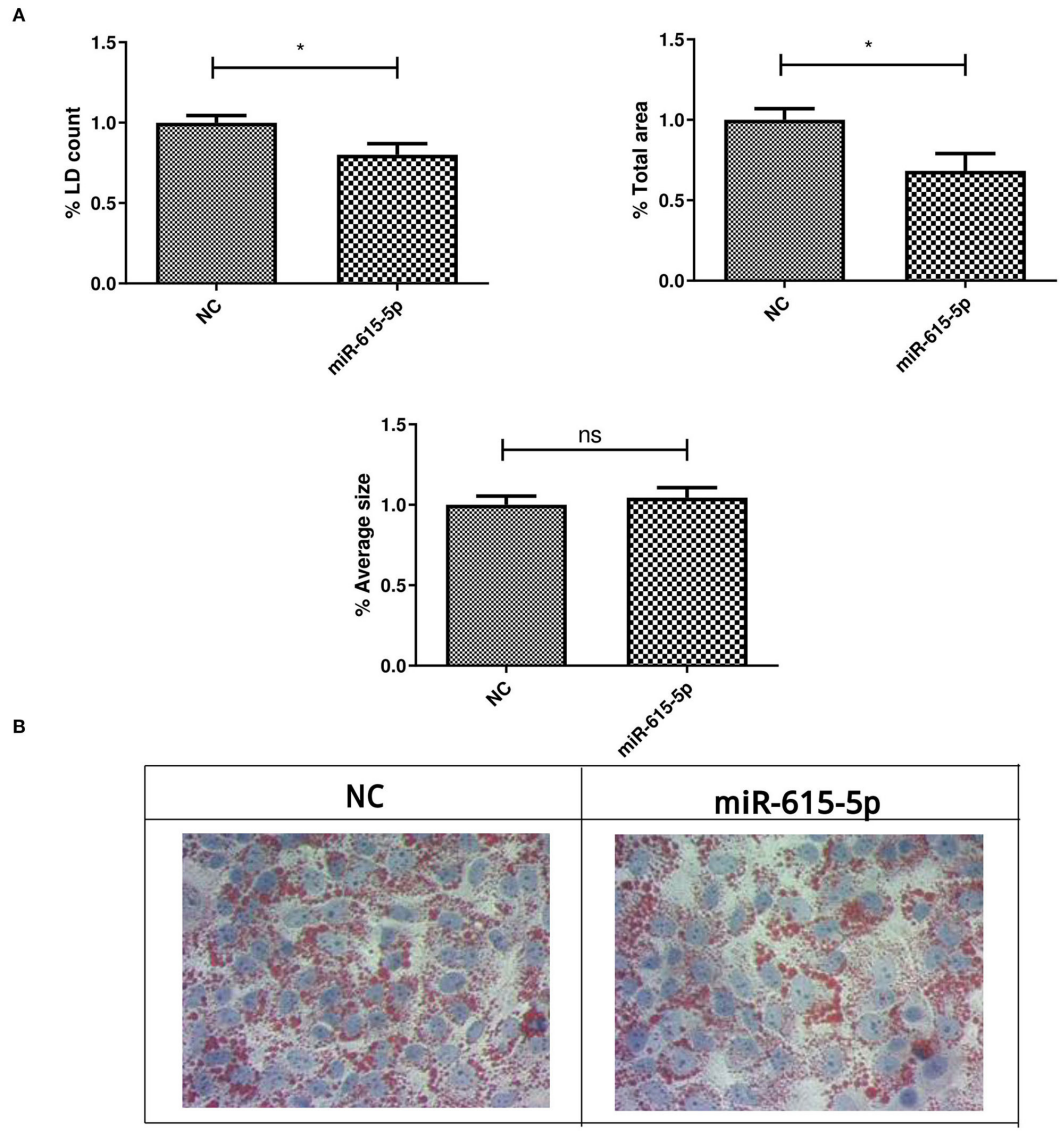
LD characterization after treating Huh-7 cells with an miR-615-5p mimic after OA treatment. LD characterization in Huh-7 cells treated with 800 μM OA after manipulation of miR-615-5p. **(A)** miR-615-5p showed a significant decrease in the %LD count (*p* = 0.0162) (left panel) and total area (*p* = 0.0158) (right panel); however, no significant change was detected on the average size of LD (*p* = 0.5848) (lower panel) compared to NC (*n* = 3, duplicate). **(B)** Representative figure from three independent experiments showing a decrease in the LD count upon miR-615-5p mimicking in Huh-7 cells treated with OA compared to NC. The unpaired two-tailed *t*-test was performed. *P < 0.05, and ns, statistically not significant.

Lipid droplets were characterized in Huh-7 cells treated with 800 μM OA, followed by transfection of miR-615-5p mimics. Treatment with miR-615-5p showed a significant decrease in the LD count (*p* = 0.0162, 0.8002 ± 0.06855) compared to NC (1.000 ± 0.04403), as well as total area (*p* = 0.0158, 0.6833 ± 0.1070) compared to NC (1.000 ± 0.06993). However, no significant change was detected in the average size of LDs (*p* = 0.5848) compared to NC (*n* = 3, duplicate) (Figures 7A,B).

### Impact of miR-615-5p over-expression in Huh-7 cells treated with 800 μM OA on triglyceride accumulation

**Figure 8:**
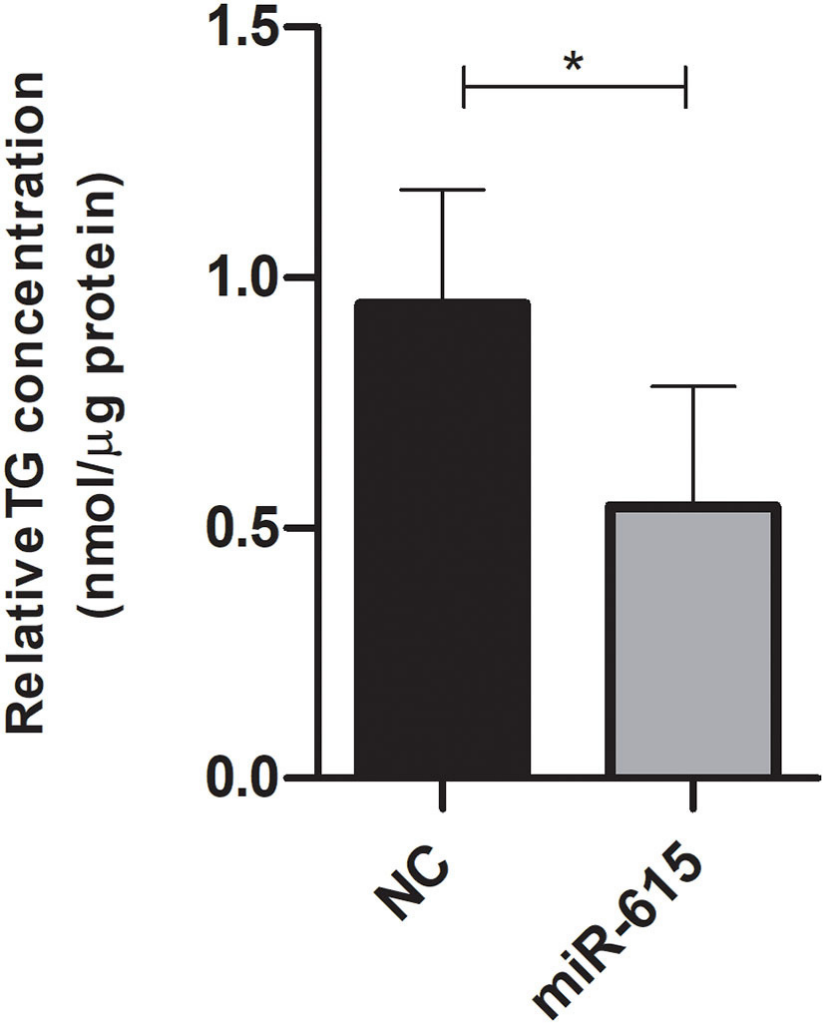
Triglyceride quantification after treatment with miR-615-5p mimics after OA treatment. Relative triglyceride quantification in Huh-7 cells treated with 800 μM OA after forcing the expression of either miR-615-5p or NC. miR-615-5p mimics showed a significant decrease in TG (*p* = 0.0108) compared to NC (*n* = 3). For each sample, the TG concentration was normalized to its respective protein level. The paired two-tailed *t*-test was performed. *P < 0.05, and ns, statistically not significant.

Triglycerides were extracted and quantified from Huh-7 cells treated with 800 μM OA after transfection with miR-615-5p mimics or NC oligonucleotides. Cells mimicked with miR-615-5p showed decreased TG (*p* = 0.0108) compared to NC (*n* = 3) (Figure 8).

### Impact of *H19* on mTOR and SREBP1c mRNA levels in Huh-7 cells treated with 800 μM OA

**Figure 9:**
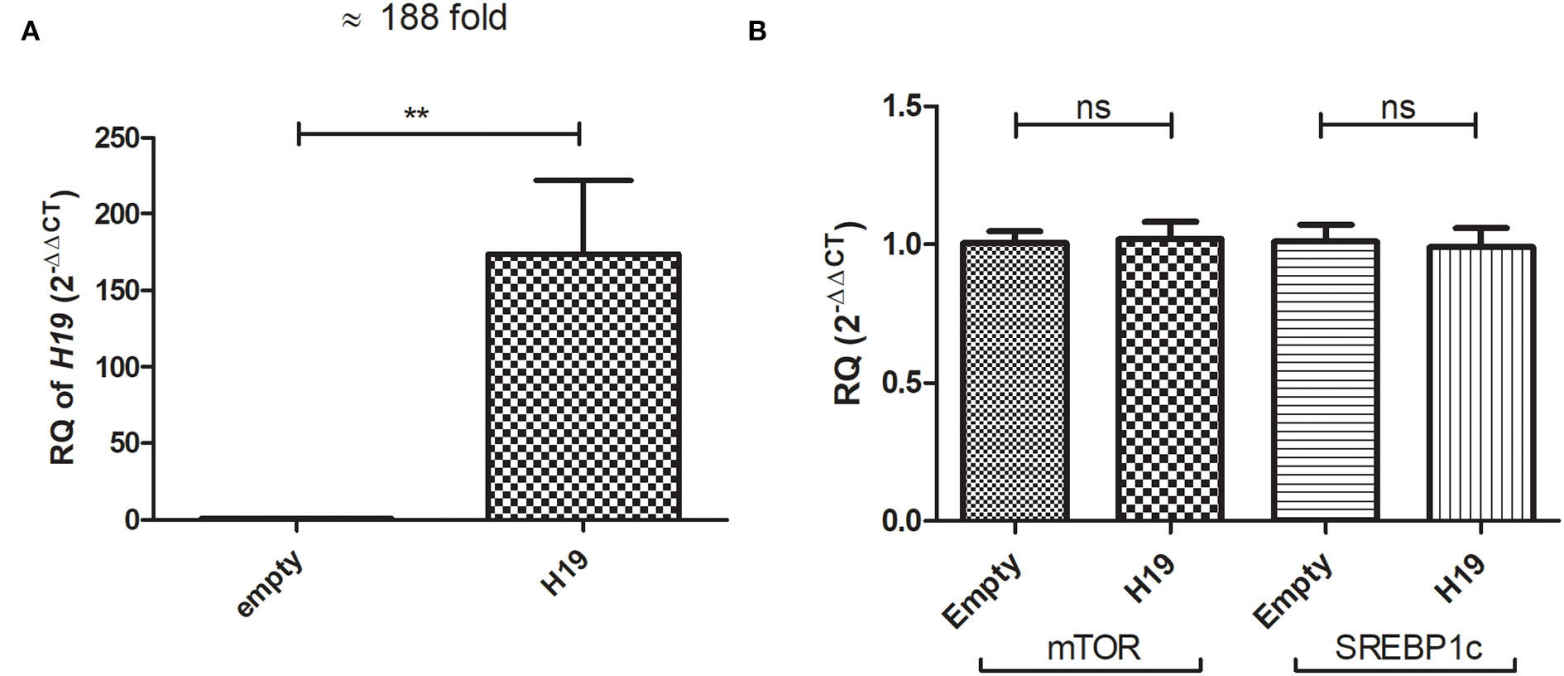
Impact of *H19* on mTOR and SREBP1c in Huh-7 cells treated with 800 μM OA. The relative expression of *H19* was determined in Huh-7 cells treated with 800 μM OA 48 h post-*H19* over-expression. The *H19* vector elevated its expression with a mean 188-fold increase (*p* = 0.0051) compared to cells transfected with the empty vector **(A)**. Relative mRNA expression of mTOR (*p* = 0.8498) and SREBP1c (*p* = 0.8405) showed no significant difference between cells over-expressing *H19* compared to the empty vector (*n* > 3, duplicate) **(B)**. The unpaired two-tailed *t*-test was performed. **P < 0.01.

The Huh-7 cells were treated with 800 μM OA, and the next day, lncRNA *H19* over-expression or empty vectors were transfected. Over-expression was first confirmed, and *H19* was over-expressed with a mean 188-fold increase (*p* = 0.0024) (173.9 ± 48.04) compared to empty vector (1.018 ± 0.07986) (Figure 9A). The relative expression of mTOR and SREBP1c mRNA levels was determined in the Huh-7 cells treated with 800 μM OA, after *H19* over-expression. Upon forcing the expression of *H19*, no significant change was observed in either mTOR (*p* = 0.8498) (1.020 ± 0.06345) or SREBP1c (*p* = 0.8405) (0.9917 ± 0.06840) relative mRNA expression compared to the empty vector (1.005 ± 0.04274) (1.011 ± 0.06120), respectively (*n* > 3, duplicate) (Figure 9B).

### Impact of *H19* on mTOR and SREBP1c protein levels in Huh-7 cells treated with 800 μM OA

**Figure 10:**
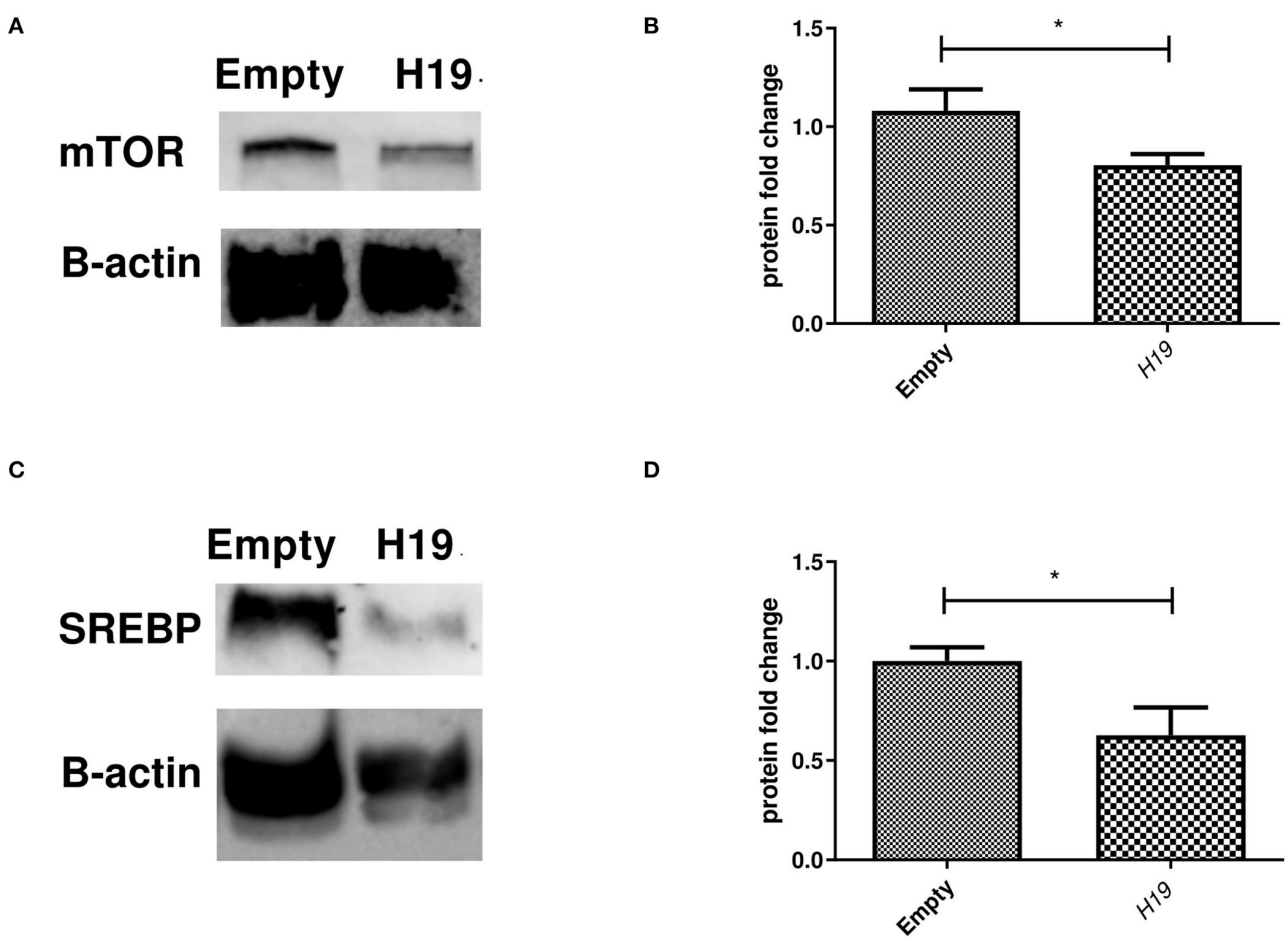
Impact of forcing the expression of *H19* on mTOR and SREBP1 protein level. Impact of *H19* on the mTOR/SREBP axis at the protein level. **(A)** Representative figure of mTOR blots showing a decrease in the mTOR protein level following *H19* over-expression. **(B)** Relative quantification of mTOR protein levels in cells treated with the *H19* over-expression vector compared to the empty vector (*n* = 3) (*p* = 0.0379). **(C)** Representative figure of SREBP1c blots showing reduced SREBP1c protein levels in cells treated with *H19* compared to the empty vector. **(D)** Relative quantification of SREBP1c in cells over-expressing *H19* compared to the empty vector (*p* = 0.0332) (*n* > 3). The unpaired two-tailed *t*-test was performed. *P < 0.05.

Inducing the expression of *H19* in the Huh-7 cells treated with 800 μM OA resulted in a decrease in the mTOR protein level (*p* = 0.0379, 0.8042 ± 0.05646) compared to the empty vector (1.081 ± 0.1089) (*n* = 3) (Figures 10A,B). At the same time, *H19* decreased SREBP1c protein levels (*p* = 0.0332, 0.6267 ± 0.1406) compared to the empty vector (1.000 ± 0.06994) (*n* > 3) (Figures 10C,D).

### Impact of *H19* over-expression in Huh-7 cells treated with 800 μM OA on lipid droplets

**Figure 11:**
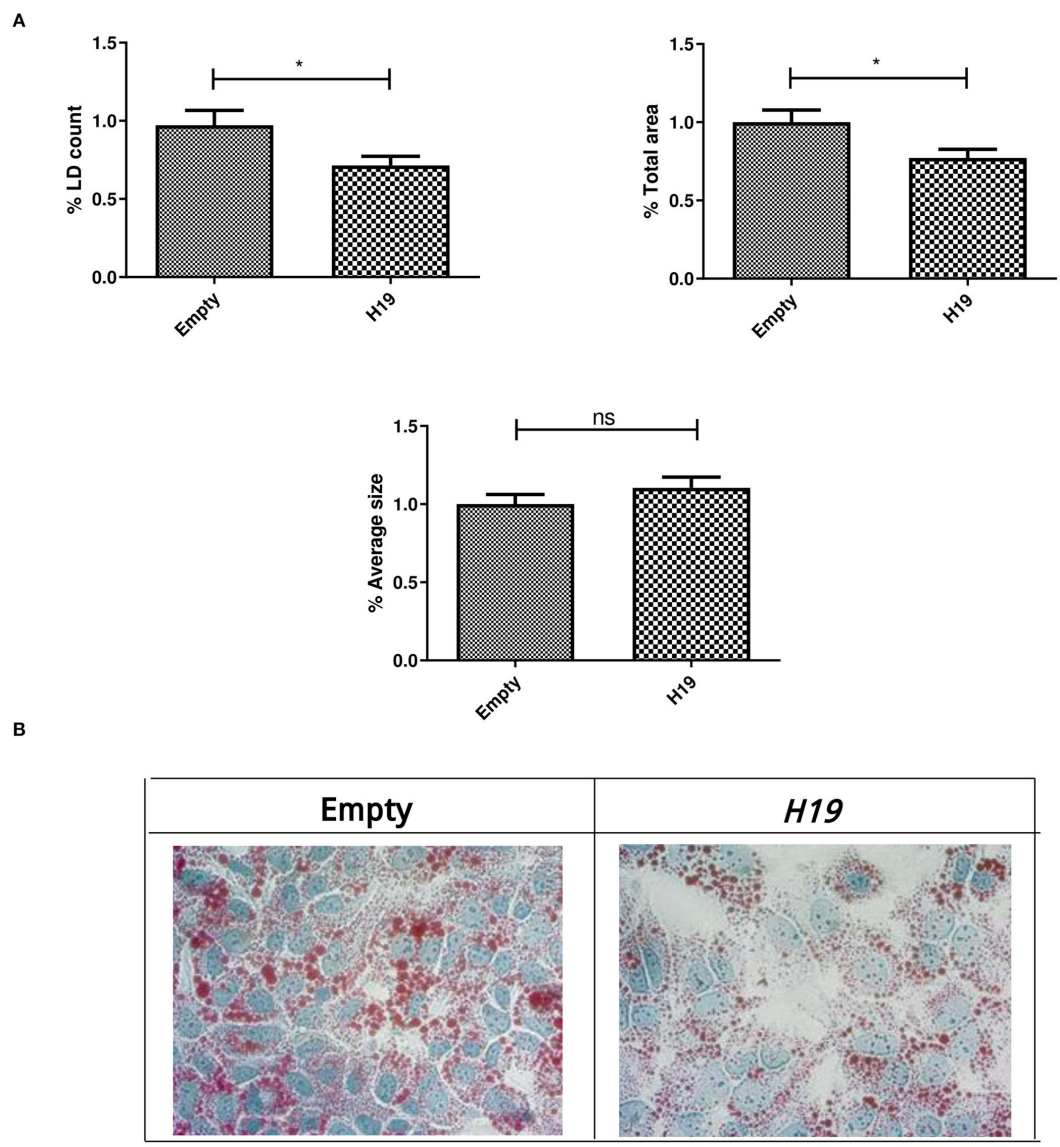
LD characterization after treatment with *H19* over-expression vector. LD characterization in Huh-7 cells treated with 800 μM OA after forcing the expression of *H19*. **(A)** H19 showed a significant decrease in the %LD count (*p* = 0.0164) (left panel) and total area (*p* = 0.0226) (right panel); however, no significant difference in average size was observed (*p* = 0.1536) (lower panel) compared to the empty vector (*n* > 3, duplicate). **(B)** Representative figure from three independent experiments showing the a decrease in the LD count upon *H19* induction in Huh7 cells treated with OA compared to the empty vector. The unpaired two-tailed *t*-test was performed. *P < 0.05, and ns, statistically not significant.

Lipid droplets were characterized in the Huh-7 cells treated with 800 μM OA, followed by transfection of the *H19* over-expression vector or empty vector. *H19* over-expression showed a significant decrease in the LD count (*p* = 0.0164) and total area (*p* = 0.0226) but had no significant effect on average size (*p* = 0.1536) compared to the empty vector (*n* > 3, duplicate) (Figures 11A,B).

### Impact of *H19* over-expression in Huh-7 cells treated with 800 μM OA on triglyceride accumulation

**Figure 12:**
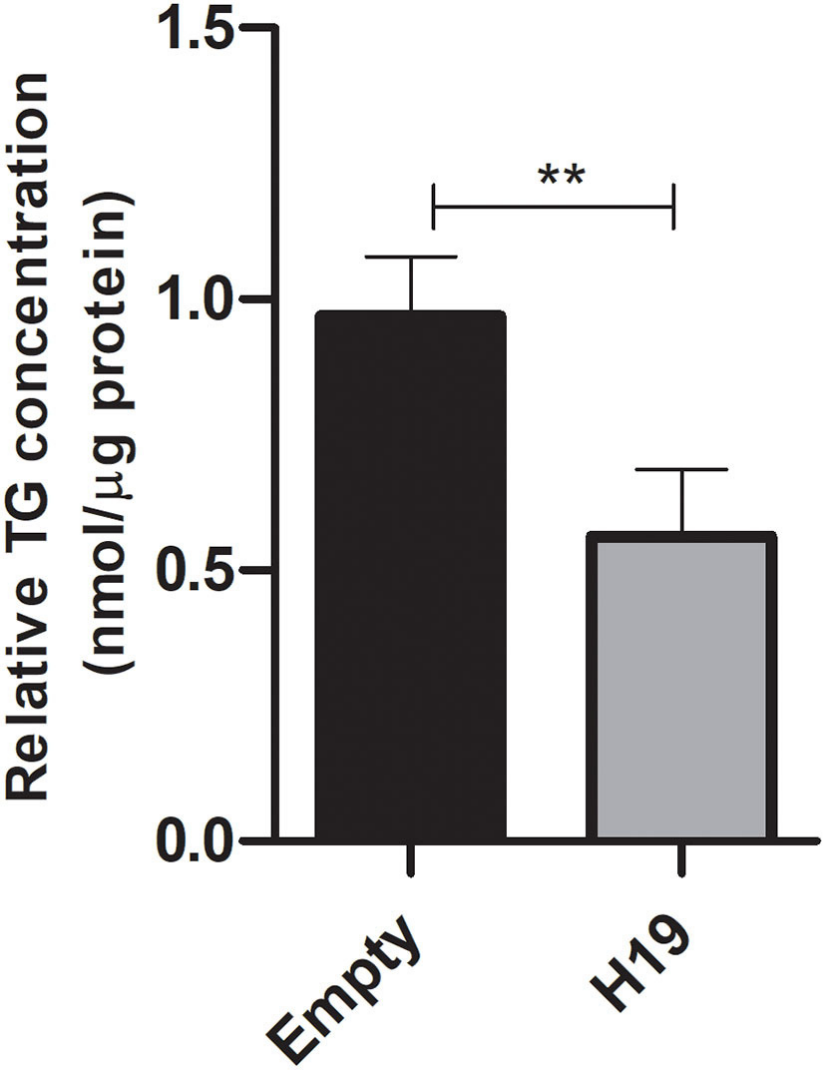
Triglyceride quantification after treatment with *H19* over-expression vector. Triglyceride quantification in Huh-7 cells treated with 800 μM OA after forcing the expression of *H19* over-expression or the empty vector. *H19* over-expression showed a significant decrease in TG (*p* = 0.0012) compared to the empty vector (*n* = 3). For each sample, the TG concentration was normalized to its respective protein level. The paired two-tailed *t*-test was performed. **P < 0.01, and ns, statistically not significant.

Triglycerides were extracted and quantified from Huh-7 cells treated with 800 μM OA after transfection with *H19* over-expression or empty vector. Cells over-expressing *H19* showed decreased TG levels compared to cells transfected with the empty vector (*p* = 0.0012) (*n* = 3) (Figure 12).

## Discussion

NAFLD is characterized by the main hallmark of TG accumulation, which can be achieved by either increased TG influx into the liver or increased DNL and thus TG synthesis, or decreased export or hydrolysis of TG (27). Therapeutic approaches involving molecular targeting of critical events leading to NAFLD have been recently investigated. Such approaches include targeting pro-lipogenic enzymes and nuclear receptors related to impaired lipogenic signaling. This was supported by the finding that knocking down or knocking out of key enzymes needed for fatty acid (FA) synthesis in steatotic mice models showed reversal of metabolic defects associated with the condition (28, 29). Since FA synthesis is controlled by lipogenic transcription factors, our aim was to study the regulation of the main lipogenic transcription factor responsible for DNL, SREBP1c. We sought to achieve this by targeting its upstream regulator, mTOR, using both short and long ncRNAs, thus regulating TG accumulation and LD formation. This was of particular interest as LDs are increasingly showing roles in various other diseases in addition to steatosis, such as HCV infection and several cancers (30–32).

To induce LD formation, Huh-7 cells were treated with 800 μM OA, the concentration of fasting plasma-free FA of patients with NASH (23). To ensure that this concentration was not toxic to Huh-7 cells, viability was assessed using different concentrations of OA ranging from 250 to 1,000 μM. Up to 1,000 μM OA showed no sign of toxicity in Huh-7 cells at both 24 and 48 h (Figures 1A,B); on the contrary, increased viability was observed. This is in line with the study by Chafez-Tapia et al. who also showed the absence of cytotoxicity of palmitic/oleic acid 1:2 molar ratio up to 1,200 μM (33). It is also in agreement with the study by Park et al. (34) who showed increased proliferation of HepG2 cell lines upon OA treatment. Nevertheless, this opposes the finding of Giulitti et al. (35) who reported decreased proliferation of liver cancer cell lines upon OA treatment; however, this effect was not observed in healthy liver-derived human cell lines. This discrepancy regarding the effect of OA on cell viability and proliferation might be attributed to the different methods used to prepare OA, different OA doses used, or OA treatment for different time intervals. However, the viability experiment was mainly performed to exclude that the 800 μM OA preparation used in subsequent experiments is toxic. To validate that this OA concentration promotes LD formation, LDs were stained with oil red-O, where significantly increased oil red-O absorbance confirmed the success of LD induction (Figure 2). Accordingly, this treatment fulfills the following criteria: (1) it simulates fasting levels of free FAs in patients with NASH, (2) it is not toxic to the cells and (3) it induces LD formation.

Dietary fat was shown to upregulate SREBP (36). In addition, SREBP was found to be elevated in animal models with fatty liver and insulin resistance; moreover, despite insulin resistance in peripheral tissues, insulin was still able to activate SREBP in the liver, thus inducing downstream lipogenic enzymes (37, 38). Accordingly, we were highly interested in studying the regulation of SREBP1c (the main TF for DNL) through targeting its upstream regulator mTOR by short and long ncRNAs. mTOR can activate lipogenesis in energy-abundant fed states (13) as it promotes SREBP1c expression and maturation (14, 39); this can partially explain the selective insulin resistance underlying enhanced lipogenesis in obese mice (37, 40).

To regulate mTOR and thus SREBP1c, two ncRNAs, miR-615-5p and *H19*, were chosen. miR-615-5p was previously characterized by our group as a tumor suppressor and shown to target mTOR, but to our knowledge, this was never investigated before in fatty liver (18, 41). On the other hand, long ncRNA *H19* was found to inhibit mTORC1 in pituitary tumors (20). The role of *H19* in lipid metabolism is controversial. When PLIN2, a member of the lipid droplet protein family, was inhibited, this was associated with a 548-fold increase in *H19* levels accompanied by decreased liver TGs, suggesting a role for *H19* in steatosis (12). However, despite decreasing precursor SREBP1c protein levels, *H19* was reported to stabilize nuclear SREBP1c, thus promoting lipid accumulation in high-fat or fasting state *in vitro* and *in vivo* studies (42). Despite the controversy, a role for *H19* in lipid metabolism and mTORC1 regulation is apparent.

Accordingly, it was interesting to measure the baseline levels of these ncRNAs and the lipogenic transcription factors upon OA treatment. miR-615-5p was shown, for the first time, to be significantly upregulated after OA treatment (Figure 3A). On the other hand, *H19* was downregulated (Figure 3B). This is in line with the finding of Khaoula et al. who showed a decreased expression of lncRNA *H19* in an HepG2 steatotic model (43). However, our findings oppose the result of Liu et al. which reported an induction of *H19* upon FA treatment. It is worth noting that the latter study used 200 μM OA treatment for only 16 hours. This might show how dose, as well as time of exposure, can affect the cell transcriptomic response and highlights the complexity of generating a fatty liver model (42). Nonetheless, the altered expression of both miR-615-5p and lncRNA *H19* in OA-treated cells could lead to significant changes in their downstream target genes.

Interestingly, both mTOR and SREBP1c showed reduced mRNA expression upon OA treatment (Figure 4). The low level of SREBP1c is in line with the fact that polyunsaturated FAs can induce SREBP mRNA degradation (44). In addition, FAs were shown to antagonize the effect of agonists of LXR, an upstream inducer of SREBP1c (45, 46). Our findings are also in line with the study by van Deursen et al. who reported decreased SREBP2 activity by oleate (44, 47, 48). However, this contradicts another study by Rojas et al., although this discrepancy might be attributed to differences in cell lines used or OA preparation methods (49). In addition, in the context of our findings, the downregulation of both mTOR and SREBP might also be explained by the increased levels of miR-615-5p, which we found in OA-treated cells. Hence, the impact of miR-615-5p on these transcription factors was examined.

Forcing the expression of miR-615-5p caused a significant concomitant decrease in the mRNA levels of both mTOR and SREBP1c (Figure 5). This impact was also examined on the translational level, where both mTOR and SREBP1c protein levels were dramatically reduced by miR-615-5p (Figure 6). This is in agreement with our previous study that showed miR-615-5p decreased mTOR levels in Huh-7 cells (18). Since miR-615-5p showed a suppressive effect on the mTOR/SREBP axis, it was intriguing to investigate its subsequent impact on LDs. Indeed, miR-615-5p was able to decrease the LD count and total LD area without an impact on average LD size (Figure 7), showing, for the first time, a potential role for miR-615-5p in regulating LDs. Similarly, miR-615-5p reduced TG accumulation (Figure 8). Interestingly, its counterpart, miR-615-3p, has demonstrated a role in reversing lipotoxicity (50), which may suggest a function for the miR-615 duplex in alleviating fatty liver. Taken together, the anti-lipogenic activity of miR-615-5p, along with its ability to reduce both mTOR and SREBP1c, supports that co-regulation of these two transcription factors has a potential role in lowering lipogenesis. This was also proved by Wang et al. who showed that suppressing both mTOR and SREBP—as opposed to suppressing only SREBP—was more efficient in lowering LDs and lipogenesis in diabetic kidneys both *in vivo* and *in vitro* (51).

The effect of ncRNA *H19* on regulating the mTOR/SREBP axis was also explored. No impact was found on either mTOR or SREBP1c mRNA levels upon inducing *H19* expression (Figure 9). Liu et al. also showed that *H19* had no effect on the SREBP1c mRNA level (42). Since lncRNAs can mediate their action translationally, rather than on the transcription level, the impact of forcing the expression of *H19* on the protein levels of mTOR and SREBP1c was assessed. Indeed, *H19* was able to reduce the protein levels of both mTOR and SREBP1c (Figure 10). This opposes the study by Liu et al. that reported increased stability of SREBP1 by *H19* in cells treated with OA. Again, these discrepant findings may be due to the differences in experimental setups. The exact mechanism by which *H19* regulates the TF on the protein, rather than the mRNA, level requires further elucidation.

Since *H19* affected the protein levels of both mTOR and SREBP, it was interesting to determine its impact on LDs, especially since its role in lipid regulation has been controversial. In fact, *H19* over-expression was able to reduce the LD count and total LD area with no effect on average LD size (Figure 11). As further confirmation, *H19* over-expression reduced TG accumulation (Figure 12). This is in consensus with the study by Imai et al. who reported decreased TG levels in the presence of high *H19* levels, as well as Gao et al. who showed that TG levels in muscle cells were decreased upon *H19* induction (12, 52). However, Liu et al. reported increased LDs upon *H19* transduction using AAV8 virus. This might be partially explained by the differences in the experimental design as *H19* was first transduced in Huh-7 cells, then 72 h later, the cells were treated with 200 μM OA for 16 h (42). In this study, the cells were treated with 800 μM OA, transfected the next day with *H19* over-expression plasmids, and then 48 h later, LDs and TG were characterized. Thus, the impact of *H19* might be time-dependent or OA dose-dependent.

In light of these findings, miR-615-5p is shown for the first time as a novel regulator of the mTOR/SREBP axis on both the mRNA and protein levels, with an impact on LDs and TG accumulation. In addition, *H19* exhibited a functional role on LDs and TG accumulation, which may be partially explained by its ability to reduce mTOR and SREBP protein levels, also suggesting a role of *H19* in regulating the same axis. It is important to note that *in vivo* and *in vitro* models for NAFLD that recapitulate the exact NAFLD metabolic hallmarks are still hard to achieve to date (53–55). However, our findings can be considered as first steps in regulating LD formation through ncRNAs, which remains to be further confirmed in different NAFLD models. In addition, it would be of great interest to study genes downstream to SREBP1 to further elucidate and validate the role of miR-615 or lncRNA *H19* in LD and TG regulation. Our findings are especially interesting after the success of the ARO-HSD clinical trial. ARO-HSD is the first RNA interfering (RNAi) therapeutic agent that knocks down hydroxysteroid 17-beta dehydrogenase 13 (HSD17B13) mRNA and protein levels and protects patients with NASH with acceptable tolerability (56). Accordingly, regulation of the mTOR/SREBP1c axis with ncRNAs provides a promising NASH therapeutic approach.

## Data availability statement

The original contributions presented in the study are included in the article/supplementary material, further inquiries can be directed to the corresponding author/s.

## Author contributions

AA conceived and supervised the study. SE and NA performed the experiments. NE-E, IF, and NE co-supervised the work process. NE provided new tools and reagents. SE, NE-E, IF, and AA analyzed the data. SE wrote the manuscript. AA made the manuscript revisions. All authors contributed to the article and approved the submitted version.

## Funding

Part of this study is based upon work supported by the Egyptian Science, Technology and Innovation Funding Authority, under Young Researcher Grant (YRG-43240).

## Conflict of interest

The authors declare that the research was conducted in the absence of any commercial or financial relationships that could be construed as a potential conflict of interest.

## Publisher's note

All claims expressed in this article are solely those of the authors and do not necessarily represent those of their affiliated organizations, or those of the publisher, the editors and the reviewers. Any product that may be evaluated in this article, or claim that may be made by its manufacturer, is not guaranteed or endorsed by the publisher.
